# Twisted benign ovarian teratoma presenting with pain and generalized pruritus: a case report

**DOI:** 10.1186/1752-1947-7-130

**Published:** 2013-05-13

**Authors:** Raeed Deen, Asantha de Silva, Ruwan Wijesuriya

**Affiliations:** 1 Department of Biology, Cornell University, Ithaca, NY 14850, USA; 2 Department of Surgery, University of Kelaniya Medical School, PO Box 6, Talagolla Road, Ragama, Sri Lanka

## Abstract

**Introduction:**

Cystic ovarian teratomas comprise 20% of all ovarian neoplasms, and are commonly encountered in patients between 20 and 40 years of age. Although these cysts are usually asymptomatic, we present the case of a patient whose cyst resulted in pruritus and abdominal pain. Based on a MEDLINE search of the literature, we believe this is the first case report of a twisted ovarian cyst presenting with generalized pruritus.

**Case presentation:**

A 35-year-old Sri Lankan woman presented with lower abdominal pain of one day’s duration with vomiting and generalized pruritus. She had no history of allergies and was not on medication. Upon a physical examination, our patient was found to have an acute abdomen, localized peritonism in her lower abdomen and tachycardia of 100 beats per minute. Computed tomography showed that the cyst, which contained calcified structures, originated from her left ovary. After laparoscopy-assisted removal of the twisted ovarian cyst, her symptoms resolved completely. Histological examination confirmed a benign ovarian teratoma.

**Conclusions:**

An unusual case of torsion of an ovarian teratoma presenting with abdominal pain and generalized pruritus, believed to be due to an antibody-mediated response, was resolved after surgical removal of the cyst.

## Introduction

Mature cystic ovarian teratomas comprise 20% of all ovarian neoplasms and are encountered in the second or third decade of life [1]. These cysts are usually asymptomatic and are identified incidentally during either physical or radiological examination of the abdomen, usually for unrelated reasons. We present an unusual case of a twisted ovarian teratoma in a woman who presented as an emergency with an acute abdomen and generalized pruritus.

## Case presentation

A 35-year-old Sri Lankan woman, previously well, presented with lower abdominal pain of one day’s duration with vomiting and generalized pruritus. She did not report a history of previous allergies and was not on medication at the time of presentation. An examination revealed an acute abdomen, localized peritonism in her lower abdomen and tachycardia of 100 beats per minute. Hematological investigations revealed a normal full blood count and normal serum amylase. A chest radiograph excluded free intraperitoneal gas. An ultrasound scan of her abdomen showed a thick-walled cyst in her pelvis of uncertain origin and a plain computed tomography showed that the cyst, with calcified structures, originated from her left ovary.

On laparoscopic examination under general anesthesia, we found a cyst attached to her left ovary, which had twisted. After clipping and disconnecting its pedicle from her ovary, we attempted to aspirate its contents to facilitate extraction through a laparoscopic 10mm port. Because the contents of the cyst were too viscid, we could not achieve a reduction in cyst size through aspiration and the cyst was finally extracted through a 5cm transverse supra-pubic incision.

**Figure 1:**
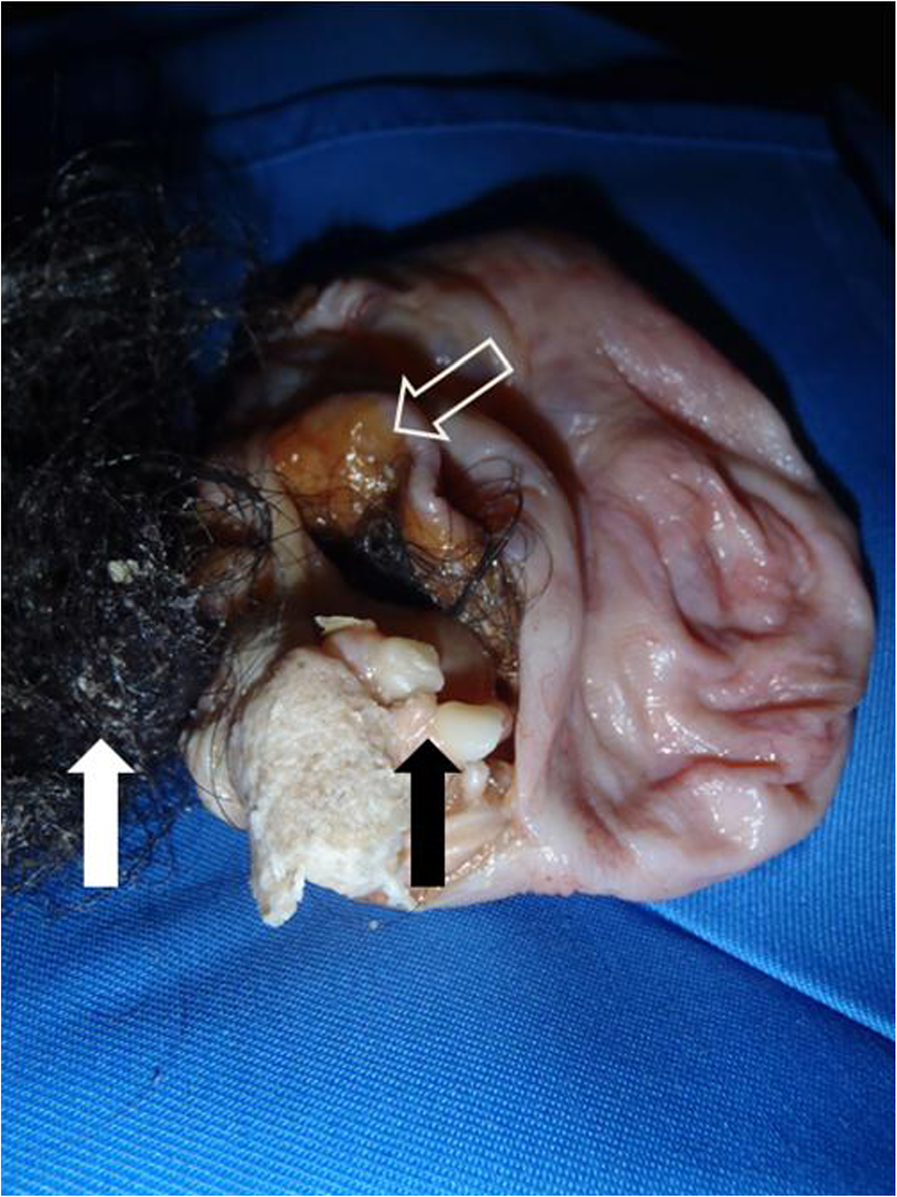
The contents of the removed cyst revealed rudimentary calcific teeth (solid black arrow), hair (solid white arrow) and sebaceous content (white outlined arrow).

Our patient recovered well after surgery and was discharged home on the first postoperative day, free of her generalized pruritus. Bisection of the cyst wall showed that it contained 200mL of thick, offensive milky brown fluid, hair and rudimentary teeth (Figure 1). Histological analysis of the cyst wall revealed that it was a benign teratoma.

## Discussion

Our patient presented as an emergency due to torsion of the cyst on its long pedicle. In this regard, generalized pruritus likely arose from cyst wall ischemia and release of antigenic content into the general circulation, as has been demonstrated previously [2]. Because the onset of pruritus correlated with her abdominal pain arising from torsion of the cyst, and her pruritus resolved after cyst removal, it is likely that her pruritus was an antibody-mediated response to the contents of her cyst. In addition to sebaceous content, we also encountered hair and rudimentary teeth in the cyst (Figure 1). These arose from totipotent cells in the teratoma, which is known to comprise one or more germ cell layers.

We searched MEDLINE using the key words ‘ovarian teratoma’, ‘torsion’, ‘pruritus’ and ‘itching’, and to the best of our knowledge believe that no similar previous case report exists.

## Conclusions

An unusual case of a woman with torsion of an ovarian teratoma presenting with abdominal pain and generalized pruritus, believed due to an antibody-mediated response, was resolved after surgical removal of the cyst. The cyst was found to contain sebaceous material, rudimentary teeth and hair.

### Consent

Written informed consent was obtained from the patient for publication of this case report and accompanying images. A copy of the written consent is available for review by the Editor-in-Chief of this journal.

## Competing interests

The authors declare that they have no competing interests.

## Authors’ contributions

RD wrote and revised several drafts of the manuscript and photographed the cyst. AdS conceptualized the case report and reviewed the manuscript. RW performed the surgery and reviewed the manuscript. All authors read and approved the final manuscript.

## References

[B1] Ozgur T Atik E Silfeler DB Toprak S Mature cystic teratomas in our series with review of the literature and retrospective analysis Arch Gynecol Obstet 2012 285 1099 1101 10.1007/s00404-011-2171-8 22167448

[B2] Tuzun E Zhou L Baehring JM Bannykh S Rosenfield MR Dalmau J Evidence for antibody-mediated pathogenesis in anti-NMDAR encephalitis associated with ovarian teratoma Acta Neuropathol 2009 118 737 743 10.1007/s00401-009-0582-4 19680671 PMC2888642

